# Microwave quantum illumination using a digital receiver

**DOI:** 10.1126/sciadv.abb0451

**Published:** 2020-05-08

**Authors:** S. Barzanjeh, S. Pirandola, D. Vitali, J. M. Fink

**Affiliations:** 1 Institute of Science and Technology Austria, 3400 Klosterneuburg, Austria.; 2 Department of Computer Science, University of York, Deramore Lane, York YO10 5GH, UK.; 3 Research Laboratory of Electronics, Massachusetts Institute of Technology, Cambridge, MA 02139, USA.; 4 School of Science and Technology, Physics Division, University of Camerino, Camerino (MC), Italy.; 5 INFN, Sezione di Perugia, Perugia Italy.; 6 CNR-INO, Florence, Italy.

## Abstract

Quantum illumination uses entangled signal-idler photon pairs to boost the detection efficiency of low-reflectivity objects in environments with bright thermal noise. Its advantage is particularly evident at low signal powers, a promising feature for applications such as noninvasive biomedical scanning or low-power short-range radar. Here, we experimentally investigate the concept of quantum illumination at microwave frequencies. We generate entangled fields to illuminate a room-temperature object at a distance of 1 m in a free-space detection setup. We implement a digital phase-conjugate receiver based on linear quadrature measurements that outperforms a symmetric classical noise radar in the same conditions, despite the entanglement-breaking signal path. Starting from experimental data, we also simulate the case of perfect idler photon number detection, which results in a quantum advantage compared with the relative classical benchmark. Our results highlight the opportunities and challenges in the way toward a first room-temperature application of microwave quantum circuits.

## Teaser

A quantum radar prototype shows how quantum entanglement could help detect objects in noisy ambient conditions.

## INTRODUCTION

Quantum sensing is well developed for photonic applications (*1*) in line with other advanced areas of quantum information (*2*–*5*). Quantum optics has been, so far, the most natural and convenient setting for implementing the majority of protocols in quantum communication, cryptography, and metrology (*6*). The situation is different at longer wavelengths, such as tetrahertz or microwaves, for which the current variety of quantum technologies is more limited and confined to cryogenic environments. With the exception of superconducting quantum processing (*7*), no microwave quanta are typically used for applications such as sensing and communication. For these tasks, high-energy and low-loss optical and telecom frequency signals represent the first choice and form the communication backbone in the future vision of a hybrid quantum internet (*8*–*10*).

Despite this general picture, there are applications of quantum sensing that are naturally embedded in the microwave regime. This is exactly the case with quantum illumination (QI) (*11*–*17*) for its remarkable robustness to background noise, which, at room temperature, amounts to ∼10^3^ thermal quanta per mode at a few gigahertz. In QI, the aim is to detect a low-reflectivity object in the presence of very bright thermal noise. This is accomplished by probing the target with less than one entangled photon per mode, in a stealthy noninvasive fashion, which is impossible to reproduce with classical means. In the Gaussian QI protocol (*12*), the light is prepared in a two-mode squeezed vacuum state (*3*) with the signal mode sent to probe the target, while the idler mode is kept at the receiver. Although entanglement is lost in the round trip from the target, the surviving signal-idler correlations, when appropriately measured, can be strong enough to beat the performance achievable by the most powerful classical detection strategy. In the low photon flux regime, where QI shows the biggest advantage, it could be suitable for extending quantum sensing techniques to short-range radar (*18*) and noninvasive diagnostic scanner applications (*19*).

Previous experiments in the microwave domain (*20*, *21*) demonstrated a quantum enhancement of the detected covariances compared with a symmetric classical noise radar, i.e., with approximately equal signal and idler photon number. With appropriate phase-sensitive detection, an ideal classically correlated noise radar can be on par or, in the case of a bright idler (*17*), even outperform coherent heterodyne detection schemes, which maximize the signal-to-noise ratio (SNR) for realistic (phase-rotating) targets. However, if the phase of the reflected signal is stable over relevant time scales or a priori known, then homodyne detection represents the strongest classical benchmark.

**Fig. 1:**
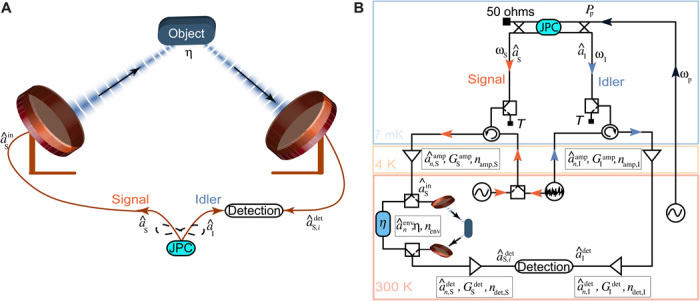
Implementation of microwave QI. (**A**) Schematic representation of microwave QI. A quantum source generates and emits stationary entangled microwave fields in two separate paths. The signal mode ${\hat{a}}_{S}$ is used to interrogate the presence (*i* = 1) or absence (*i* = 0) of a room-temperature object with total roundtrip reflectivity η. The returned mode ${\hat{a}}_{S,i}^{\text{det}}$ is measured together with the unperturbed idler mode ${\hat{a}}_{I}$. (**B**) Circuit diagram of the experimental setup. A superconducting Josephson parametric converter (JPC) is used to entangle signal and idler modes at frequencies ω_S_ and ω_I_ by applying a suitable parametric pump tone at the sum frequency ω_p_ = ω_S_ + ω_I_ at ∼ 7 mK. A coherent microwave tone or a classically correlated noise source is used to generate benchmark signals at room temperature that are sent into the dilution refrigerator and reflected from the JPC ports. The outputs of the JPC or the reflected classical signals are amplified, down converted, and digitized simultaneously and independently for both channels. The signal mode passes through a measurement line that contains a room-temperature switch that is used to select between a digitally controllable attenuator η and a free-space link realized with two antennas and a movable reflective object. Here, we consider η as the total signal loss between the two room temperature switches used in our measurement chain. For the system noise and gain calibration, we use two latching microwave switches at cold temperatures, which are used to select between the JPC outputs and a temperature *T* variable 50-ohm load (black squares). In both panels above, the final detection step corresponds to a two-channel quadrature measurement, followed by digital postprocessing.

In this work, we implement a digital version of the phase-conjugate receiver of (*22*), experimentally investigating proof-of-concept QI in the microwave regime (*23*). We use a Josephson parametric converter (JPC) (*24*, *25*) inside a dilution refrigerator for entanglement generation (*26*, *27*). The generated signal microwave mode, with annihilation operator ${\hat{a}}_{S}$, is amplified to facilitate its detection and sent to probe a room-temperature target, while the idler mode ${\hat{a}}_{I}$ is measured as schematically shown in Fig. 1A. The reflection from the target ${\hat{a}}_{R}$ is also detected, and the two measurement results are postprocessed to calculate the SNR for discriminating the presence or absence of the object. Our experimental implementation of QI relies on linear quadrature measurements and suitable postprocessing to compute all covariance matrix elements from the full measurement record, as shown in previous microwave quantum optics experiments with linear detectors (*28*–*30*). This enables an implementation of the phase-conjugate receiver that fully exploits the correlations of the JPC output fields without analog photodetection. We then compare the SNR with other detection strategies for the same signal path, i.e., the same signal photon numbers at the JPC output, which is also our reference point for the theoretical modeling.

Our digital approach to QI circumvents common practical problems such as finite idler storage time that can limit the range and fidelity of QI detection schemes. However, this advantage comes at the expense that the theoretically strongest classical benchmark in the same conditions—the coherent-state homodyne detector using the same signal power and signal path—can be approached in specific conditions such as quantum-limited amplification, but never be outperformed. To outperform coherent-state homodyne detection in practice, we will require low-temperature square law detection of microwave fields that can be realized with radiometer or photon counting measurements. Nevertheless, using calibration measurements of the idler path, we can simulate a situation with perfect idler photon number detection, extrapolating the case where the reflected mode is detected together with the idler mode using analog microwave photon counters. For this situation, we show that the SNR of coherent heterodyne detection and symmetric noise radars is exceeded by up to 4 dB and that of homodyne detection—the classical benchmark—by up to 1 dB for the same amplified signal path, measurement bandwidth, and signal power. We also note that the strong and noisy amplification of the signal path chosen to facilitate the detection with commercial analog-to-digital converters enables another classical receiver strategy, i.e., the detection of the amplifier noise in the presence of the target. Since the amplified noise exceeds the environmental noise at room temperature by orders of magnitude, this would be the most effective strategy for the implemented experiment. For the same reason, a low-noise coherent source at room temperature would outperform the relative benchmarks considered here. In practice, outperforming the room temperature benchmark depends on the chosen amount of gain, the type of amplifier, and the loss in the detection system and therefore does not pose a fundamental limitation to the presented measurement scheme that focuses on the relative comparison of the different illumination types.

## RESULTS

The experimental setup, shown in Fig. 1B, is based on a frequency tunable superconducting JPC operated in the three-wave mixing regime and pumped at the sum of signal and idler frequencies ω_p_ = ω_S_ + ω_I_; see Materials and Methods for more details. The output of the JPC contains a nonzero phase-sensitive cross-correlation $\langle {\hat{a}}_{S}{\hat{a}}_{I}\rangle$, which leads to entanglement between the signal mode with frequency ω_S_ = 10.09 GHz and the idler mode with frequency ω_I_ = 6.8 GHz. In our work, the quantities $\langle \hat{O}\rangle$ and ${\left(\Delta O_{i}\right)}^{2}=\langle {\hat{O}}_{i}^{2}\rangle -{\langle {\hat{O}}_{i}\rangle }^{2}$ define the mean and the variance of the operator $\hat{O}$, respectively, and they are evaluated from experimental data. The signal and idler are sent through two different measurement lines, where they are amplified, filtered, down converted to an intermediate frequency of 20 MHz, and digitized with a sampling rate of 100 MHz using an 8-bit analog-to-digital converter. Applying fast Fourier transform (FFT) and postprocessing to the measured data, we obtain the quadrature voltages *I_i_* and *Q_i_*, which are related to the complex amplitudes *a_i_* and their conjugate $a_{i}^{*}$ of the signal and idler modes at the outputs of the JPC as $a_{i}=\frac{I_{i}+i\;Q_{i}}{\sqrt{2\hbar \omega_{i}BRG_{i}}}$ and $a_{i}^{*}=\frac{I_{i}-i\;Q_{i}}{\sqrt{2\hbar \omega_{i}BRG_{i}}}$, having the same measurement statistics as the annihilation operator ${\hat{a}}_{i}$. Here, *R* = 50 ohms, *B* = 200 kHz is the measurement bandwidth set by a digital filter, and *i* = S, I (*30*–*32*). We calibrate the system gain (*G*_S_, *G*_I_) = (93.98(01),94.25(02)) dB and system noise (*n*_add, S_, *n*_add, I_) = (9.61(04),14.91(1)) of both measurement channels as described in Materials and Methods.

**Fig. 2:**
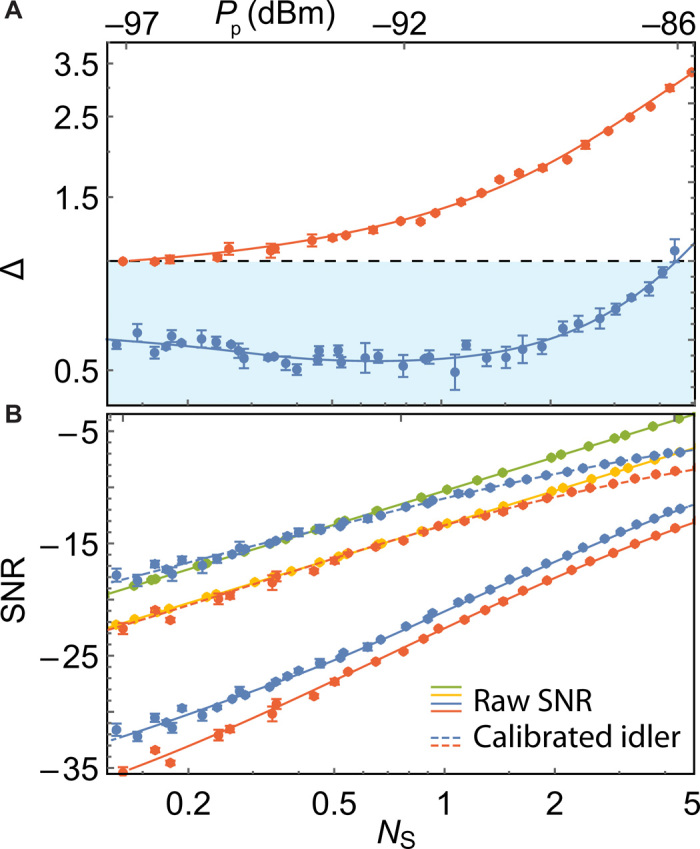
Entanglement and QI. (**A**) The measured entanglement parameter Δ for the output of the JPC (blue) and classically correlated noise (orange) as a function of the inferred signal photon number *N*_S_ at the output of the JPC and the pump power *P*_p_ at the input of the JPC. (**B**) Comparison of the measured single-mode signal-to-noise ratio (SNR) of QI (solid blue), symmetric classically correlated illumination (CI, solid orange), coherent-state illumination with homodyne (solid green) and heterodyne detection (solid yellow), and the inferred SNR of calibrated QI (dashed blue) and CI (dashed orange) as a function of the signal photon number *N*_S_ for a perfectly reflective object and a 5-μs measurement time. The dots are measured and inferred data points, and the solid and dashed lines are the theory prediction. For both (A) and (B), the error bars indicate the 95% confidence interval based on three sets of measurements, each with 380,000 two-channel quadrature pairs for QI/CI, and 192,000 quadrature pairs for coherent-state illumination.

A first important check for the experiment is to quantify the amount of entanglement at the output of the JPC at 7 mK. A sufficient condition for the signal and idler modes to be entangled is the nonseparability criterion $\Delta ≔\langle {\hat{X}}_{-}^{2}\rangle +\langle {\hat{P}}_{+}^{2}\rangle <1$ (*33*), for the joint field quadratures ${\hat{X}}_{-}=({\hat{a}}_{S}+{\hat{a}}_{S}^{†}-{\hat{a}}_{I}-{\hat{a}}_{I}^{†})/2$ and ${\hat{P}}_{+}=({\hat{a}}_{S}-{\hat{a}}_{S}^{†}+{\hat{a}}_{I}-{\hat{a}}_{I}^{†})/(2i)$. In Fig. 2A, we show measurements of Δ as a function of the signal photon number $N_{S}=\langle {\hat{a}}_{S}^{†}{\hat{a}}_{S}\rangle$ at the output of the JPC at millikelvin temperatures, as obtained by applying the above calibration procedure to both signal and idler modes, and compare the result with classically correlated radiation. The latter is generated at room temperature using the white noise mode of an arbitrary waveform generator, divided into two different lines, individually up-converted to the signal ω_S_ and idler ω_I_ frequencies, and fed to the JPC inside the dilution refrigerator. Note that, for both JPC and classically correlated noise, we digitally rotate the relative phase of the quadratures to maximize the correlation between signal and idler.

The classically correlated signal and idler modes are then reflected back from the JPC (pumps are off) and pass through the measurement lines attached to the outputs of the JPC. This ensures that both classical and quantum radiations experience the same conditions in terms of gain, loss, and noise before reaching the target and before being detected in the identical way. As shown in Fig. 2A, at low photon number, the parameter Δ is below one, proving that the outputs of the JPC are entangled, while at larger photon number (larger pump power), the entanglement gradually degrades and vanishes at *N*_S_ = 4.5 photons s^−1^ Hz^−1^. We attribute this to finite losses in the JPC, which leads to pump power–dependent heating and results in larger variances of the output field. The classically correlated radiation of the same signal power, on the other hand (orange data points), cannot fulfill the nonseparability criterion, and therefore, Δ ≥ 1 for the entire range of the signal photons. In the latter case, we also observe a slow relative degradation of the classical correlations as a function of the signal photon numbers, which could be improved with more sophisticated noise generation schemes (*20*).

The experiments of QI and classically correlated illumination (CI) are implemented in a similar way (see Fig. 1B). The two amplified quadratures of the idler mode ${\hat{a}}_{I}^{\text{det}}$ are measured at room temperature, and the signal mode ${\hat{a}}_{S}$ is amplified (with gain $G_{S}^{\text{amp}}$ and the noise mode ${\hat{a}}_{n,S}^{\text{amp}}$) and used to probe a noisy region that is suspected to contain an object. In this process, we define η as the total detection loss on the signal path between the two room-temperature switches used in the measurement chain, which includes cable loss, free-space loss, and object reflectivity. The reflected signal from the region is measured by means of a mixer and an amplifier with gain $G_{S}^{\text{det}}$ and the noise mode ${\hat{a}}_{n,S}^{\text{det}}$. The output ${\hat{a}}_{S,i}^{\text{det}}$ in the presence (*i* = 1) or absence (*i* = 0) of the object is then postprocessed for the reconstruction of the covariance matrix of the detected signal-idler state.

The signal mode ${\hat{a}}_{S,i}^{\text{det}}$ takes different forms depending on the presence

$$\begin{array}{c} {\hat{a}}_{S,1}^{\text{det}}=\sqrt{G_{S}}(\sqrt{\eta }{\hat{a}}_{S}+\sqrt{\frac{\eta (G_{S}^{\text{amp}}-1)}{G_{S}^{\text{amp}}}}{\hat{a}}_{n,S}^{\text{amp}†}+\\ \sqrt{\frac{1-\eta }{G_{S}^{\text{amp}}}}{\hat{a}}_{n}^{\text{env}}+\sqrt{\frac{G_{S}^{\text{det}}-1}{G_{S}}}{\hat{a}}_{n,S}^{\text{det}†}) \end{array}$$ (1)

or absence

$${\hat{a}}_{S,0}^{\text{det}}=\sqrt{G_{S}^{\text{det}}}({\hat{a}}_{n}^{\text{env}}+\sqrt{1-\frac{1}{G_{S}^{\text{det}}}}{\hat{a}}_{n,S}^{\text{det}†})$$ (2)

of the target with ${\hat{a}}_{n}^{\text{env}}$ as the environmental noise mode. In the absence of the object, the signal contains only noise $n_{0}=G_{S}^{\text{det}}n_{\text{env}}+(G_{S}^{\text{det}}-1)n_{\text{det},S}$ in which *n*_det, S_ is the amplifier-added noise after interrogating the object region. In the presence of the target and for η ≪ 1, the added noise to the signal is $n_{1}=\eta G_{S}^{\text{det}}(G_{S}^{\text{amp}}-1)n_{\text{amp},S}+n_{0}$, whose first term is due to the amplifier added noise of the first amplification stage before reaching the target, which exceeds the environmental noise *n*_env_ as well as the signal photon numbers used to probe the target.

This implies that, in our proof-of-principle demonstration, the optimal classical strategy would actually be based on detecting the presence or absence of the amplifier noise rather than the coherences and correlations of the signal-idler path with the measured SNR_passive_ = (*n*_1_ − *n*_0_)/(*n*_0_ + 1) ≃ 31.4 dB for the chosen gain and receiver noise in our setup. However, for lower-noise-temperature signal amplifiers and lower gain, as well as in longer range applications with increased loss, such a passive signature of the detection scheme will be markedly reduced and eventually disappear in the environmental noise at room temperature.

The final step of the measurement is the application of a digital version of the phase-conjugate receiver (*22*). The reflected mode ${\hat{a}}_{S,i}^{\text{det}}$ is first phase conjugated and then combined with the idler mode on a 50:50 beam splitter. As described in Materials and Methods, the SNR of the balanced difference photodetection measurement reads

$${\text{SNR}}_{\text{QI}/\text{Cl}}=\frac{{\left(\langle {\hat{N}}_{1}\rangle -\langle {\hat{N}}_{0}\rangle \right)}^{2}}{2{\left(\sqrt{{\left(\Delta N_{1}\right)}^{2}}+\sqrt{{\left(\Delta N_{0}\right)}^{2}}\right)}^{2}}$$ (3)

where ${\hat{N}}_{i}={\hat{a}}_{i,+}^{†}{\hat{a}}_{i,+}-{\hat{a}}_{i,-}^{†}{\hat{a}}_{i,-}$ with ${\hat{a}}_{i,\pm }=({\hat{a}}_{S,i}^{\text{det}†}+\sqrt{2}{\hat{a}}_{v}\pm {\hat{a}}_{I}^{\text{det}})/\sqrt{2}$ is the annihilation operator of the mixed signal and idler modes at the output of the beam splitter in the absence (*i* = 0) and the presence (*i* = 1) of the target (here, ${\hat{a}}_{v}$ is the vacuum noise operator). For the raw SNR without idler calibration, we use Eq. 3. To simulate perfect photon number detection of the idler mode directly at the JPC output, we reduce the variance in the denominator of Eq. 3 by the calibrated idler vacuum and amplifier noise as $\langle {\hat{a}}_{I}^{†}{\hat{a}}_{I}\rangle =\langle {\hat{a}}_{I}^{\text{det}†}{\hat{a}}_{I}^{\text{det}}\rangle /G_{I}-(n_{\text{add},I}+1)$ (see Materials and Methods).

The experiment of coherent-state illumination is performed by generating a weak coherent tone using a microwave source at room temperature, followed by a low-temperature chain of thermalized attenuators inside the dilution refrigerator. The center frequency of the coherent tone is ω_S_, exactly matched with the frequency of the signal used in the QI and CI experiments. The coherent tone is reflected back from the unpumped JPC and directed into the same measurement chain identical to that of QI and CI (see Fig. 1B). The signal is sent to probe a target region, and the detected radiation ${\hat{a}}_{S,i}^{\text{det}}$ is used to calculate the SNR of the digital homodyne and heterodyne detections for the same probe power, bandwidth, and amplifier noise.

In the absence of a passive signature due to signal noise amplification, digital homodyne detection of a coherent state represents the optimal classical strategy in terms of the SNR, which is given by

$${\text{SNR}}_{\text{CS}}^{\text{hom}}=\frac{{\left(\langle {\hat{X}}_{S,1}^{\text{det}}\rangle -\langle {\hat{X}}_{S,0}^{\text{det}}\rangle \right)}^{2}}{2{\left(\sqrt{{\left(\Delta X_{S,1}^{\text{det}}\right)}^{2}}+\sqrt{{\left(\Delta X_{S,0}^{\text{det}}\right)}^{2}}\right)}^{2}}$$ (4)

while the SNR of the digital heterodyne detection is lower and given by

$${\text{SNR}}_{\text{CS}}^{\text{het}}=\frac{{\left(\langle {\hat{X}}_{S,1}^{\text{det}}\rangle -\langle {\hat{X}}_{S,0}^{\text{det}}\rangle \right)}^{2}+{\left(\langle {\hat{P}}_{S,1}^{\text{det}}\rangle -\langle {\hat{P}}_{S,0}^{\text{det}}\rangle \right)}^{2}}{2{\left(\sqrt{{\left(\Delta X_{S,1}^{\text{det}}\right)}^{2}+{\left(\Delta P_{S,1}^{\text{det}}\right)}^{2}}+\sqrt{{\left(\Delta X_{S,0}^{\text{det}}\right)}^{2}+{\left(\Delta P_{S,0}^{\text{det}}\right)}^{2}}\right)}^{2}}$$ (5)

where ${\hat{X}}_{S,i}^{\text{det}}=\frac{{\hat{a}}_{S,i}^{\text{det}}+{\hat{a}}_{S,i}^{\text{det}†}}{\sqrt{2}}$ and ${\hat{P}}_{S,i}^{\text{det}}=\frac{{\hat{a}}_{S,i}^{\text{det}}-{\hat{a}}_{S,i}^{\text{det}†}}{i\sqrt{2}}$ are the field quadrature operators (see Materials and Methods for more details).

In Fig. 2B, we compare the SNR of QI and CI with and without idler calibration for a perfectly reflective object in a zero loss channel η = 1. For comparison, we also include the results of coherent-state illumination with homodyne and heterodyne detection. In all cases, the signal mode at room temperature is overwhelmed with amplifier noise. We use three sets of measurements to calculate the SD of the mean SNR of a single mode measurement with measurement time *T* = 1/*B* = 5 μs. Each set is based on *M* = 380,000 samples (192,000 for the coherent-state detection), corresponding to a measurement time of 1.87 s (0.93 s for the coherent-state detection). To get the total statistics, the measurement time takes 5.6 s (2.8 s for the coherent-state detection). For the same measurement bandwidth and using the raw data of the measured quadrature pairs (solid lines), QI (blue dots) outperforms suboptimum symmetric CI (orange dots) by up to 3 dB at low signal photon numbers, but it cannot compete with the SNR obtained with coherent-state illumination (yellow and green dots). Under the assumption of perfect idler photon number detection, i.e., applying the calibration discussed above (dashed lines), the SNR of QI is up to 4 dB larger than that of symmetric CI and coherent-state illumination with heterodyne detection, which does not require phase information, over the region where the outputs of the JPC are entangled. For signal photon numbers *N*_S_ > 4.5, where there is no entanglement present in the signal source, the sensitivity of the coherent-state transmitter with heterodyne detection outperforms QI and CI, confirming the critical role of entanglement to improve the sensitivity of the detection.

QI with a phase-conjugate receiver is potentially able to outperform coherent-state illumination with homodyne detection by up to 3 dB, i.e., the optimum classical benchmark, in the regime of low signal photon numbers. In the region *N*_S_ < 0.4, the experimentally inferred SNR of QI is approximately 1 dB larger, in agreement with the theoretical prediction taking into account experimental nonidealities like the finite squeezing of the source. In practice though, i.e., without the applied idler calibration, the quantum advantage compared with coherent homodyne detection is not accessible with a digital receiver based on heterodyne measurements, even in the case of quantum limited amplifiers, due to the captured idler vacuum noise, which lowers the optimal SNR by at least 3 dB (*12*, *16*). The experimental results (dots) are in very good agreement with the theoretical prediction (solid and dashed lines). For the theory, we rewrite the SNRs Eqs. 3 to 5 in terms of the signal photon number $N_{S}=\langle {\hat{a}}_{S}^{†}{\hat{a}}_{S}\rangle$, the idler photon number $N_{I}=\langle {\hat{a}}_{I}^{†}{\hat{a}}_{I}\rangle$, and the signal-idler correlation $\langle {\hat{a}}_{S}{\hat{a}}_{I}\rangle$ at the output of the JPC. These parameters are extracted from the measured and calibrated data as a function of the JPC pump power. Together with the known system gain and noise, we plot the theoretical predictions of the various protocols at room temperature.

**Fig. 3:**
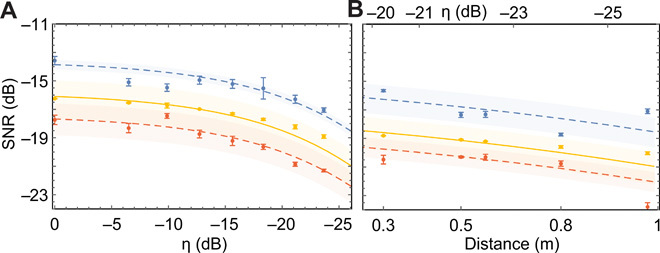
Low-reflectivity quantum correlated noise radar. The inferred SNR of calibrated QI (blue) and symmetric CI (orange), and the measured coherent-state illumination with digital heterodyne detection (yellow) as a function of (**A**) the total signal loss η and (**B**) object distance from the transmitting and receiving antennas for free-space illumination. The error bars are calculated similar to Fig. 2. For both (A) and (B), the signal photon number is *N*_S_ = 0.5. The shaded regions are the theoretical uncertainties extracted by fitting the experimental data. The SNR of the coherent state with homodyne detection is not presented in this figure since the expected advantage at the chosen *N*_S_ is smaller than systematic errors in this measurement.

An important feature of a radar or short-range scanner is its resilience with respect to signal loss. To verify this, as shown in Fig. 1B, we use two microwave switches at room temperature in the signal line to select between a digitally controllable step attenuator to mimic an object with tunable reflectivity and a proof-of-principle radar setup. With this setup, we determine the effects of loss and object reflectivity as well as target distance on the efficiency of the quantum enhanced radar. In Fig. 3A, we plot the measured SNR of QI, CI, and coherent-state illumination with heterodyne detection as a function of the imposed loss on the signal mode. The calibrated QI protocol is always superior to calibrated symmetric CI and coherent-state illumination with heterodyne detection for a range of effective loss −25 dB <η < 0 dB. The dashed lines are the theory predictions from Eqs. 3 and 5 for a fixed chosen signal photon number *N*_S_ = 0.5. The shaded regions represent the confidence interval extracted from the SD of the measured idler photon numbers, and the cross-correlations as a function of η.

In the context of radar, small improvements in the SNR lead to the exponentially improved error probability $ℰ=1/2\text{erfc}\;(\sqrt{\text{SNR}\cdot M})$, where *M* = *T*_tot_*B* is the number of single mode measurements, and *T*_tot_ is the total measurement time required for a successful target detection. To test the principle of microwave QI in free space at room temperature, we amplify and send the microwave signal emitted from the JPC to a horn antenna and a copper plate representing the target at a variable distance. The reflected signal from this object is collected using a second antenna of the same type, down converted, digitized, and combined with the calibrated idler mode to calculate the SNR of the binary decision. With this setup, we repeat the measurement for CI and coherent-state illumination with heterodyne. Figure 3B shows the SNR of these protocols as a function of the object distance from the transmitting antenna as well as the total loss of the free space link. Calibrated QI reveals higher sensitivity for a reflective target up to 1 m away from the transmitting antenna. The results are in good agreement with the theoretical model.

## DISCUSSION

In this work, we have studied proof-of-concept QI in the microwave domain, the most natural frequency range for target detection. Assuming perfect idler photon number detection, we showed that a quantum advantage is possible despite the entanglement-breaking signal path. Since the best results are achieved for less than one mean photon per mode, our experiment indicates the potential of QI as a noninvasive scanning method, e.g., for biomedical applications, imaging of human tissues, or nondestructive rotational spectroscopy of proteins, besides its potential use as short-range low-power radar, e.g., for security applications. However, for this initial proof-of-principle demonstration, the amplified bright noise in the target region overwhelms the environmental noise by orders of magnitude, which precludes the noninvasive character at short target distances and presents an opportunity to use the presence or absence of the amplifier noise to detect the object with even higher SNR. The use of quantum-limited parametric amplifiers (*34*–*36*) with limited gain, such that the amplified vacuum does not significantly exceed environmental or typical electronic noise at the target, will help to achieve a practical advantage with respect to the lowest-noise-figure coherent-state heterodyne receivers at room temperature, and, up to the vacuum noise, they will also render the idler calibration obsolete. The use of sensitive radiometers or microwave single-photon detectors (*37*–*39*), at millikelvin temperatures without signal amplification, represents a promising route to achieve an advantage in practical situations and with respect to ideal coherent-state homodyne receivers. One advantage of the presented digital implementation of QI is that it does not suffer from the idler storage problem of receivers that rely on analog photodetection schemes, inherently limiting the accessible range when used as a radar. It is an interesting open question what other types of receivers (*40*) could be implemented in the microwave domain, based on the state-of-the-art superconducting circuit technology and digital signal processing.

## MATERIALS AND METHODS

### Josephson parametric converter

**Fig. 4:**
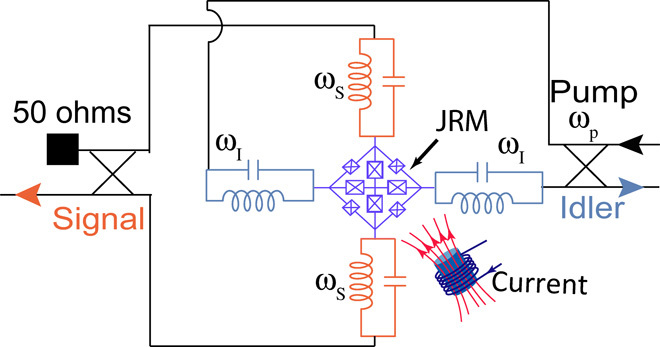
Schematic representation of the JPC. The Josephson parametric amplifier (JPC) contains a Josephson ring modulator (JRM) consisting of four Josephson junctions, and four large Josephson junctions inside the ring act as a shunt inductance for the JRM (*41*). Two microwave resonators are coupled to the JRM forming idler and signal resonators with resonance frequencies ω_I_ and ω_S_, respectively. These resonators are capacitively coupled to the input and output ports. To use the JPC in the three-wave mixing condition, the device is biased using an external magnetic field and pumped at frequency ω_p_ = ω_I_ + ω_S_. Two broadband 180° hybrids are used to feed-in and feed-out the pump, idler, and signal. In this configuration, the second port of the signal is terminated using a 50-ohm cold termination.

We use a nondegenerate three-wave mixing JPC that acts as a nonlinear quantum-limited amplifier whose signal, idler, and pump ports are spatially separated, as shown in Fig. 4. The nonlinearity of the JPC originates from a Josephson ring modulator (JRM) consisting of four Josephson junctions arranged on a rectangular ring and four large shunting Josephson junctions inside the ring (*41*). The total geometry supports two differential and one common mode. The correct bias point is selected by inducing a flux in the JRM loop by using an external magnetic field. The two pairs of the microwave half-wavelength microstrip transmission line resonators connected to the center of JRM serve as signal and idler microwave resonators. These resonators are coupled to two differential modes of the JRM and capacitively attached to two external feedlines, coupling in and out the microwave signal to the JPC.

The entanglement between signal mode with frequency ω_S_ and idler mode with frequency ω_I_ is generated by driving the nonresonant common mode of the JRM at frequency ω_p_ = ω_I_ + ω_S_. Two off-chip, broadband 180° hybrids are used to add the idler or signal modes to the pump drive. In our configuration, we apply the pump to the idler side and terminate the other port of the signal hybrid with a 50-ohm cold termination. The frequency of the signal mode is ω_S_ = 10.09 GHz, and the frequency of the idler mode is ω_I_ = 6.8 GHz. The maximum dynamical bandwidth and gain of our JPC are 20 MHz and 30 dB, respectively. The 1-dB compression point corresponds to the power −128 dBm at the input of the device at which the device gain drops by 1 dB and the amplifier starts to saturate. The frequency of the signal and idler modes can be varied over the 100-MHz span by applying a direct current to the flux line.

### Noise calibration

**Fig. 5:**
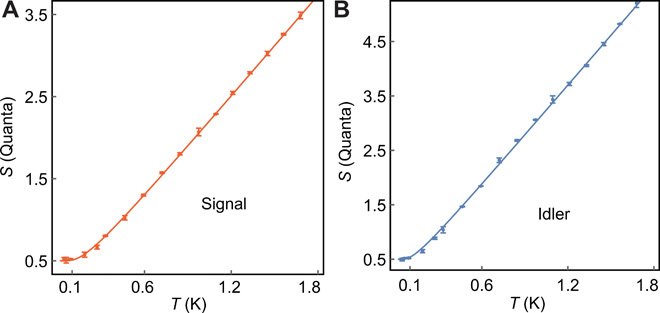
System noise calibration. Calibration of signal (**A**) and idler (**B**) output channels. The measured noise density in units of quanta, *S_i_* = *N_i_*/(ħω*_i_BRG_i_*) − *n*_add, *i*_, is shown as a function of the temperature *T* of the 50-ohm load. The error bars indicate the SD obtained from three measurements with 576,000 quadrature pairs each. The solid lines are fits to Eq. 5 in units of quanta, which yields the system gain and noise with the standard errors (95% confidence interval) as stated in section Results.

The system gain *G_i_* and system noise *n*_add, *i*_ of both signal and idler measurement chains are calibrated by injecting a known amount of thermal noise using two temperature-controlled 50-ohm cold loads (*26*, *42*). The calibrators are attached to the measurement setup with two copper coaxial cables of the same length and material as the cables used to connect the JPC via two latching microwave switches (Radiall R573423600). A thin copper braid was used for weak thermal anchoring of the calibrators to the mixing chamber plate. By measuring the noise density in V^2^/Hz at each temperature as shown in Fig. 5 and fitting the obtained data with the expected scaling

$$N_{i}={\hbar \omega }_{i}{\mathrm{BRG}}_{i}[(1/2)\text{coth}[{\hbar \omega }_{i}/(2k_{B}T)]+n_{\text{nadd},i}]$$ (6)

where *B* = 200 kHz and *R* = 50 ohms, we accurately back out the total gain

$$(G_{S},G_{I})=(93.98(01),94.25(02))\;\text{dB}$$ (7)

and the number of added noise photons referenced to the JPC output

$$\left(n_{\text{add},S},n_{\text{add},I})=(9.61(04),14.91(1)\right)$$ (8)

The 95% confidence values are taken from the standard error of the fit shown in Fig. 5.

### Measurement chain: Gain and added noise

**Fig. 6:**
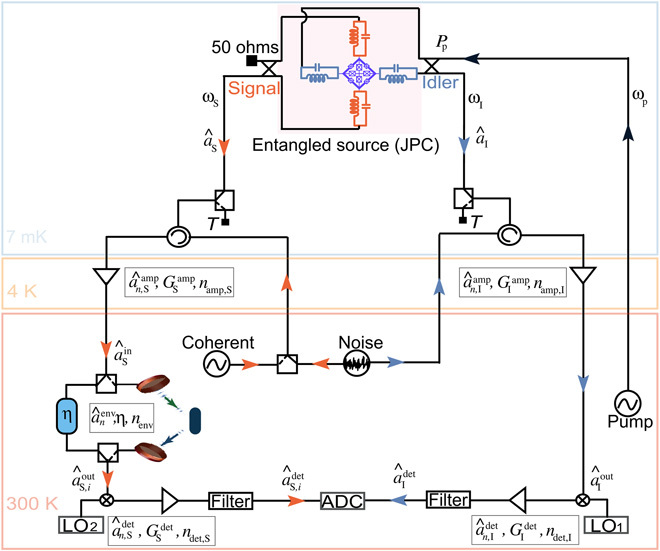
Full measurement setup. The outputs of the JPC are amplified in different stages before being down converted to 20 MHz using two local oscillators (LO_1_ and LO_2_). After the down-conversion, the signals are filtered and amplified once more and then digitized using an analog-to-digital converter (ADC). Classically CI is performed by using correlated white noise generated by an arbitrary waveform (noise) generator. For coherent-state illumination, we generate a coherent tone and send it to the refrigerator. The signal is reflected from the unpumped JPC and passes through the measurement chain.

In Fig. 6, we show the full measurement chain used in our experiment. The outputs of the JPC, the signal ${\hat{a}}_{S}$ and the idler ${\hat{a}}_{I}$, pass through two separate superconducting lines and are amplified individually using two high electron mobility transistor (HEMT) amplifiers at the 4 K temperature stage and amplified once more at room temperature. The total gain of the amplifier chain is $G_{i}^{\text{amp}}$. The output of the amplifiers for $G_{i}^{\text{amp}}\gg 1$ are

$${\hat{a}}_{S}^{\text{in}}=(\sqrt{G_{S}^{\text{amp}}}{\hat{a}}_{S}+\sqrt{G_{S}^{\text{amp}}-1}{\hat{a}}_{n,S}^{\text{amp}†})$$

$${\hat{a}}_{I}^{\text{out}}=(\sqrt{G_{I}^{\text{amp}}}{\hat{a}}_{I}+\sqrt{G_{I}^{\text{amp}}-1}{\hat{a}}_{n,I}^{\text{amp}†})$$ (9)

where ${\hat{a}}_{n,i}^{\text{amp}}$ with *i* = S, I is the annihilation operator of the noise mode added by the HEMT and one additional room temperature amplifier and the preceding cable and connector losses. The idler mode is then down converted to 20 MHz, filtered, amplified using an amplifier with gain $G_{I}^{\text{det}}$ and noise annihilation operator ${\hat{a}}_{n,I}^{\text{det}}$, and recorded using an 8-bit analog-to-digital card (ADC). The down-converted and detected idler mode is related to the idler mode right after the JPC as

$${\hat{a}}_{I}^{\text{det}}=\sqrt{G_{I}}({\hat{a}}_{I}+\sqrt{\frac{G_{I}^{\text{amp}}-1}{G_{I}^{\text{amp}}}}{\hat{a}}_{n,I}^{\text{amp}†}+\sqrt{\frac{G_{I}^{\text{det}}-1}{G_{I}}}{\hat{a}}_{n,I}^{\text{det}†})$$ (10)

where $G_{I}=G_{I}^{\text{det}}G_{I}^{\text{amp}}=94.25(02)$ dB is the total gain, and

$$\begin{array}{c} n_{\text{add},I}=\frac{G_{I}^{\text{amp}}-1}{G_{I}^{\text{amp}}}(\langle {\hat{a}}_{n,I}^{\text{amp}†}{\hat{a}}_{n,I}^{\text{amp}}\rangle +1)+\\ \frac{G_{I}^{\text{det}}-1}{G_{I}^{\text{amp}}G_{I}^{\text{det}}}(\langle {\hat{a}}_{n,I}^{\text{det}†}{\hat{a}}_{n,I}^{\text{det}}\rangle +1)=14.91(1) \end{array}$$ (11)

are the total added noise quanta referenced to the JPC output.

The signal mode is used to probe the target region. The reflected signal from the target region in the presence *H*_1_ or absence *H*_0_ of the target, respectively, is given by

$${\hat{a}}_{S,1}^{\text{out}}=\sqrt{\eta }{\hat{a}}_{S}^{\text{in}}+\sqrt{1-\eta }{\hat{a}}_{n}^{\text{env}}\;(\text{hypothesis}\;H_{1})$$ (12a)

$${\hat{a}}_{S,0}^{\text{out}}={\hat{a}}_{n}^{\text{env}}\;(\text{hypothesis}\;H_{0})$$ (12b)

where η is the total signal loss, and ${\hat{a}}_{n}^{\text{env}}$ is the annihilation operator of the environmental noise mode at room temperature. In the case of free-space illumination, we realize the absence of the target by removing the target in front of the antennas, while in the case of using a step attenuator, we mimic the absence of the target by using a 50-ohm load at the radiofrequency port of the mixer.

The signal mode after down conversion is given by

$${\hat{a}}_{S,i}^{\text{det}}=(\sqrt{G_{S}^{\text{det}}}{\hat{a}}_{S,i}^{\text{out}}+\sqrt{G_{S}^{\text{det}}-1}{\hat{a}}_{n,S}^{\text{det}†})$$ (13)

with *i* = 0,1, $G_{S}^{\text{det}}$ is the gain and ${\hat{a}}_{n,S}^{\text{det}}$ is the noise operator of the amplification stage after down conversion. Substituting Eqs. 9, 12a, and 12b into Eq. 13 gives the detected signal mode in the target presence

$$\begin{array}{c} {\hat{a}}_{S,1}^{\text{det}}=\sqrt{G_{S}}(\sqrt{\eta }{\hat{a}}_{S}+\sqrt{\frac{\eta (G_{S}^{\text{amp}}-1)}{G_{S}^{\text{amp}}}}{\hat{a}}_{n,S}^{\text{amp}†}+\\ \sqrt{\frac{1-\eta }{G_{S}^{\text{amp}}}}{\hat{a}}_{n}^{\text{env}}+\sqrt{\frac{G_{S}^{\text{det}}-1}{G_{S}}}{\hat{a}}_{n,S}^{\text{det}†}) \end{array}$$ (14)

or target absence

$${\hat{a}}_{S,0}^{\text{det}}=\sqrt{G_{S}^{\text{det}}}({\hat{a}}_{n}^{\text{env}}+\sqrt{1-\frac{1}{G_{S}^{\text{det}}}}{\hat{a}}_{n,S}^{\text{det}†})$$ (15)

where $G_{S}=G_{S}^{\text{det}}G_{S}^{\text{amp}}=93.98(01)$ dB is the total gain with $G_{S}^{\text{det}}=16.82$ dB, $G_{S}^{\text{amp}}=77.16$ dB, and

$$\begin{array}{c} n_{\text{add},S}=\frac{G_{S}^{\text{amp}}-1}{G_{S}^{\text{amp}}}(\langle {\hat{a}}_{n,S}^{\text{amp}†}{\hat{a}}_{n,S}^{\text{amp}}\rangle +1)+\\ \frac{G_{S}^{\text{det}}-1}{G_{S}^{\text{amp}}G_{S}^{\text{det}}}(\langle {\hat{a}}_{n,S}^{\text{det}†}{\hat{a}}_{n,S}^{\text{det}}\rangle +1)=9.61(04) \end{array}$$ (16)

are the total added noise quanta at the JPC output. The total added noise in the presence of the target is given by $n_{1}=\eta G_{S}^{\text{det}}(G_{S}^{\text{amp}}-1)n_{\text{amp},S}+(1-\eta )G_{S}^{\text{det}}n_{\text{env}}+(G_{S}^{\text{det}}-1)n_{\text{det},S}$, which, in the limit of η ≪ 1, leads to $n_{1}=\eta \;G_{S}^{\text{det}}(G_{S}^{\text{amp}}-1)n_{\text{amp},S}+n_{0}$, where $(G_{S}^{\text{amp}}-1)n_{\text{amp},S}\approx 5\times 10^{8}$. The total added noise in the absence of the target is $n_{0}=G_{S}^{\text{det}}n_{\text{env}}+(G_{S}^{\text{det}}-1)n_{\text{det},S}$, where $n_{\text{env}}=\langle {\hat{a}}_{n}^{\text{env}†}{\hat{a}}_{n}^{\text{env}}\rangle =672$ is the environmental thermal noise of the room-temperature object and $n_{\text{det},S}=\langle {\hat{a}}_{n,S}^{\text{det}†}{\hat{a}}_{n,S}^{\text{det}}\rangle +1\approx 3\times 10^{5}$ is the receiver noise dominated by the amplifier noise after down-conversion to the intermediate frequency.

### Digital postprocessing

**Fig. 7:**
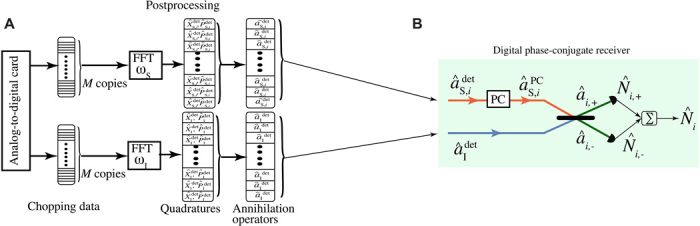
Schematic of the postprocessing. (**A**) The recorded data from the ADC is chopped in *M* shorter arrays. We apply digital FFT at idler (ω_I_) and signal frequencies (ω_S_) after analog down conversion on each array individually to infer the measurement statistics of the signal and idler mode quadratures ${\hat{X}}_{i}^{\text{det}}$ and ${\hat{P}}_{i}^{\text{det}}$ with *i* = S, I. The measurement results are then used to calculate the covariances of the signal and idler modes ${\hat{a}}_{i}^{\text{det}}=({\hat{X}}_{i}^{\text{det}}+i{\hat{P}}_{i}^{\text{det}})/\sqrt{2}$. (**B**) The digital phase-conjugate receiver used to infer the SNR of QI and CI. The *M* copies of the signal and idler modes, generated in postprocessing, are sent one by one to the digital phase-conjugate receiver. A 50:50 beam splitter mixes the phase-conjugated signal mode ${\hat{a}}_{S,i}^{\text{PC}}$ returned from the target region, with the locally detected idler mode ${\hat{a}}_{I}^{\text{det}}$. The beam splitter’s outputs are detected, yielding classical outcomes equivalent to the quantum measurements $\sum_{k=1}^{M}{\hat{N}}_{i,\pm }^{\left(k\right)}$ (includes all *M* copies), and the difference of these outputs, equivalent to the quantum measurement of ${\hat{N}}_{i}$, is used as the input to a threshold detector whose output is the target absence or presence decision.

In this section, we explain how the single-mode postprocessing was performed. As shown in Fig. 7A, the down-converted and amplified signal and idler modes are continuously recorded with 100 MS/s using a two-channel ADC with 8-bit resolution. The total measurement time of the QI/CI detections (coherent-state detections) is 5.76 s (2.88 s) in which the recorded data are chopped to *M* = 1.15 × 10^6^ (6 × 10^5^) records; each contains 500 samples, which corresponds to a filter bandwidth of 200 kHz. The 500 samples are used to perform FFT on each record individually and extract the complex quadrature voltages *I*_I_, *Q*_I_ and *I*_S_, *Q*_S_ of the intermediate frequency component at 20 MHz. We calculate the detected field quadratures of both signal and idler modes $X_{i}^{\text{det}}=I_{i}/\sqrt{\hbar \omega_{i}\mathrm{BR}}$ and $P_{i}^{\text{det}}=Q_{i}/\sqrt{\hbar \omega_{i}\mathrm{BR}}$ with *i* = S, I for *M* measurement results, which have the same measurement statistics as the quadrature operators ${\hat{X}}_{i}^{\text{det}}$ and ${\hat{P}}_{i}^{\text{det}}$, where ${\hat{a}}_{i}^{\text{det}}=({\hat{X}}_{i}^{\text{det}}+i\;{\hat{P}}_{i}^{\text{det}})/\sqrt{2}$.

### Digital phase-conjugate receiver: QI and CI

Both the JPC and a correlated classical source generate a zero-mean, two-mode Gaussian state with a nonzero cross correlation $\langle {\hat{a}}_{S}{\hat{a}}_{I}\rangle =\langle {\hat{a}}_{S}^{\text{det}}{\hat{a}}_{I}^{\text{det}}\rangle /\sqrt{G_{S}G_{I}}$. To quantify this correlation, *M* copies of the measurement results with the statistics of ${\hat{a}}_{S}^{\text{det}}$ and ${\hat{a}}_{I}^{\text{det}}$ are sent individually through the digital phase-conjugate receiver, in which we first perform phase conjugation on the received individual signal ${\hat{a}}_{S,i}^{\mathrm{PC}}=\sqrt{2}{\hat{a}}_{v}+{\hat{a}}_{S,i}^{\text{det}†}$ (${\hat{a}}_{v}$ is the vacuum operator) and then mix it with the retained corresponding idler modes on a 50:50 beam splitter, as shown in Fig. 7B, whose outputs are

$${\hat{a}}_{i,\pm }\equiv \frac{{\hat{a}}_{S,i}^{\mathrm{PC}}\pm {\hat{a}}_{I}^{\text{det}}}{\sqrt{2}}$$ (17)

The target absence-or-presence decision is made by comparing the difference of the two detectors’ total photon counts (*23*), which is equivalent to the measurement of the operator

$${\hat{N}}_{i}={\hat{N}}_{i,+}-{\hat{N}}_{i,-}$$ (18)

where ${\hat{N}}_{i,\pm }\equiv {\hat{a}}_{i,\pm }^{†}{\hat{a}}_{i,\pm }$. Since our QI protocol uses a large number of copies *M*, the central limit theorem implies that the measurement of $\sum_{k=1}^{M}{\hat{N}}_{i,\pm }^{\left(k\right)}$ yields a random variable that is Gaussian, conditioned on target absence or target presence. It follows that the receiver’s SNR for QI or CI satisfies

$${\text{SNR}}_{\text{QI}/\text{Cl}}=\frac{{\left(\langle {\hat{N}}_{1}\rangle -\langle {\hat{N}}_{0}\rangle \right)}^{2}}{2{\left(\sqrt{{\left(\Delta N_{1}\right)}^{2}}+\sqrt{{\left(\Delta N_{0}\right)}^{2}}\right)}^{2}}$$ (19)

with $\langle {\hat{O}}_{i}\rangle$ and ${\left(\Delta O_{i}\right)}^{2}=\langle {\hat{O}}_{i}^{2}\rangle -{\langle {\hat{O}}_{i}\rangle }^{2}$, for *i* = 0,1, being the conditional means and conditional variances of ${\hat{O}}_{i}$, respectively, and the brackets 〈…〉 denote an average over all of the *M* copies. For the reported raw SNR, we use Eq. 19 without any calibration applied. To infer the hypothetical SNR that could be obtained with access to the idler mode directly at the JPC output ${\hat{a}}_{I}$, we rewrite Eq. 19 in terms of single-mode moments, i.e.

$${\text{SNR}}_{\text{QI}/\text{Cl}}=\frac{{\left[(\langle {\hat{N}}_{1,+}\rangle -\langle {\hat{N}}_{1,-}\rangle )-(\langle {\hat{N}}_{0,+}\rangle -\langle {\hat{N}}_{0,-}\rangle )\right]}^{2}}{2{\left(\sqrt{{\left(\Delta N_{1}\right)}^{2}}+\sqrt{{\left(\Delta N_{0}\right)}^{2}}\right)}^{2}}$$ (20)

where

$$\langle {\hat{N}}_{0,+}\rangle -\langle {\hat{N}}_{0,-}\rangle =0$$ (21a)

$$\langle {\hat{N}}_{1,+}\rangle -\langle {\hat{N}}_{1,-}\rangle =2\sqrt{\eta \;G_{S}}\langle {\hat{a}}_{S}{\hat{a}}_{I}\rangle$$ (21b)

and (*22*)

$$\begin{array}{c} {\left(\Delta N_{i}\right)}^{2}=\langle {\hat{N}}_{i,+}\rangle (\langle {\hat{N}}_{i,+}\rangle +1)+\langle {\hat{N}}_{i,-}\rangle (\langle {\hat{N}}_{i,-}\rangle +1)-\\ {\left(\langle {\hat{a}}_{S,i}^{\mathrm{PC}†}{\hat{a}}_{S,i}^{\mathrm{PC}}\rangle -\langle {\hat{a}}_{I}^{†}{\hat{a}}_{I}\rangle \right)}^{2}/2 \end{array}$$ (22)

for *i* = 0,1, where we take the calibrated noiseless idler photon number $\langle {\hat{a}}_{I}^{†}{\hat{a}}_{I}\rangle =\langle {\hat{a}}_{I}^{\text{det}†}{\hat{a}}_{I}^{\text{det}}\rangle /G_{I}-(n_{\text{add},I}+1)$. Here, $\langle {\hat{a}}_{S}{\hat{a}}_{I}\rangle$ is presumed to be real valued, which, in general, requires phase information to apply the appropriate quadrature rotation that maximizes the signal-idler correlation.

### SNR of the coherent-state illumination: Heterodyne and homodyne measurements

To perform the coherent-state illumination, we generate a coherent signal at room temperature and send it into the dilution refrigerator, where the mode ${\hat{a}}_{S}$ is reflected at the JPC output port and passes through exactly the same measurement line, as in the case of the QI and CI protocols. The amplified signal mode is then used to probe the target region and measured via heterodyne detection. In the presence of the target, the measured signal is given by Eq. 14 with $\langle {\hat{a}}_{S,1}^{\text{det}}\rangle =\sqrt{\eta \;G_{S}}\langle {\hat{a}}_{S}\rangle$, and, in the absence of target, it is given by Eq. 15 with $\langle {\hat{a}}_{S,0}^{\text{det}}\rangle =0$. Similar to QI, we perform data processing on the recorded coherent-state outputs and use *M* measurement results of the field quadrature operators ${\hat{X}}_{S}^{\text{det}}$ and ${\hat{P}}_{S}^{\text{det}}$ to perform a digital heterodyne detection with the following SNR

$${\text{SNR}}_{\text{CS}}^{\text{het}}=\frac{{\left(\langle {\hat{X}}_{S,1}^{\text{det}}\rangle -\langle {\hat{X}}_{S,0}^{\text{det}}\rangle \right)}^{2}+{\left(\langle {\hat{P}}_{S,1}^{\text{det}}\rangle -\langle {\hat{P}}_{S,0}^{\text{det}}\rangle \right)}^{2}}{2{\left(\sqrt{{\left(\Delta X_{S,1}^{\text{det}}\right)}^{2}+{\left(\Delta P_{S,1}^{\text{det}}\right)}^{2}}+\sqrt{{\left(\Delta X_{S,0}^{\text{det}}\right)}^{2}+{\left(\Delta P_{S,0}^{\text{det}}\right)}^{2}}\right)}^{2}}$$ (23)

For the digital homodyne detection, we use phase information to rotate the signal to the relevant quadrature direction and obtain the improved SNR

$${\text{SNR}}_{\text{CS}}^{\text{hom}}=\frac{{\left(\langle {\hat{X}}_{S,1}^{\text{det}}\rangle -\langle {\hat{X}}_{S,0}^{\text{det}}\rangle \right)}^{2}}{2{\left(\sqrt{{\left(\Delta X_{S,1}^{\text{det}}\right)}^{2}}+\sqrt{{\left(\Delta X_{S,0}^{\text{det}}\right)}^{2}}\right)}^{2}}$$ (24)
